# Gut Microbiota and Sex Hormones: Crosstalking Players in Cardiometabolic and Cardiovascular Disease

**DOI:** 10.3390/ijms23137154

**Published:** 2022-06-28

**Authors:** Silvia Maffei, Francesca Forini, Paola Canale, Giuseppina Nicolini, Letizia Guiducci

**Affiliations:** 1 Department of Gynecological and Cardiovascular Endocrinology, CNR-Tuscany Region, G. Monasterio Foundation, Via G. Moruzzi 1, 56124 Pisa, Italy; silvia.maffei@ftgm.it; 2 CNR Institute of Clinical Physiology, Via G Moruzzi 1, 56124 Pisa, Italy; paola.canale95@libero.it (P.C.); nicolini@ifc.cnr.it (G.N.); letizia.guiducci@ifc.cnr.it (L.G.)

**Keywords:** gut microbiota, diet, sex hormone, cardiometabolic disease, molecular mechanisms

## Abstract

The available evidence indicates a close connection between gut microbiota (GM) disturbance and increased risk of cardiometabolic (CM) disorders and cardiovascular (CV) disease. One major objective of this narrative review is to discuss the key contribution of dietary regimen in determining the GM biodiversity and the implications of GM dysbiosis for the overall health of the CV system. In particular, emerging molecular pathways are presented, linking microbiota-derived signals to the local activation of the immune system as the driver of a systemic proinflammatory state and permissive condition for the onset and progression of CM and CV disease. We further outline how the cross-talk between sex hormones and GM impacts disease susceptibility, thereby offering a mechanistic insight into sexual dimorphism observed in CVD. A better understanding of these relationships could help unravel novel disease targets and pave the way to the development of innovative, low-risk therapeutic strategies based on diet interventions, GM manipulation, and sex hormone analogues.

## 1. Introduction

Gut microbiota (GM) is the set of microbe strains colonizing the intestinal tract. Through its genetic heritage, known as the gut microbiome, this complex ecosystem generates bioactive metabolites that impact various physiological processes, far beyond food digestion [1]. Able to communicate with distal districts through multiple pathways, GM is therefore considered the largest endocrine organ of the body and one of the major determinants of humans’ health from infancy through adulthood. Indeed, while a balanced GM facilitates beneficial effects including digestion of macronutrients, synthesis of some vitamins, maintenance of immune homeostasis, and protection against pathogens, detrimental changes in GM composition lead to adverse remodeling of the host phenotype, which predispose to several pathological conditions, such as insulin resistance, atherosclerosis, obesity, and associated disorders, ultimately leading to cardiovascular disease (CVD) [2].

Dietary habit is considered a key modifiable factor influencing the gut microflora diversity throughout one’s lifespan. Significant differences have been reported in the composition of gut microbiota between subjects fed prevalently with a western diet or a nutritional regimen rich in fibers [3]. In effect, a particular diet may favor the growth of certain bacterial strains over others, affecting fermentative metabolism and intestinal pH and promoting the development of a pathogenic flora. For example, a high-fat diet (HFD) can stimulate the expansion of a pro-inflammatory GM, with a consequent rise of circulating lipopolysaccharides (LPS) due to increased intestinal permeability. Consistently, a growing body of evidence supports the strict connections between diet, GM homeostasis, and the modulation of immune system [4].

Therefore, the GM may influence cardiometabolic (CM) risk and CVD development either directly, via metabolite production, or indirectly, by interacting with the immune system [5]. 

Besides nutrition, numerous findings on human beings and animal models point at the critical contribution of sex hormones (SH) as major regulators of the GM variability [6,7]. Interestingly, some of the bioactive molecules produced by GM have been proven to functionally interact with SH, which may explain, at least in part, the gender dimorphism of CV risks and CVD outcomes [8]. A deeper understanding of the biological processes and molecular mechanisms underlying gender differences in CM risks and CVD evolution is of pivotal importance to fine-tune differentiated strategies of prevention, diagnosis, and management of CV events.

In this review, we analyze the impact of GM, food, SH, and immune system as cross-talking, gender-related variables able to condition the onset and progression of CVD. The main objective is to provide an updated overview of the interconnected molecular mechanisms that may be effectively targeted by safe and low-cost interventions aimed at reshaping GM and preserving the homeostasis of the endocrine and immune systems with the final goal of reducing the risk for CM and CVD.

## 2. Food Intake and GM-Mediated CVD Risk

Depending on its composition, GM can transform nutrients present in the food into protective or damaging products entailing healthful or noxious effects, respectively [9]. The most representative examples of bioactive GM-derived compounds are: (1) metabolites of cholesterol such as primary bile acids (PBAs), secondary bile acids (SBAs), and coprostanol, (2) trimethylamine (TMA)/trimethylamine N-oxide (TMAO), (3) phenyl-acetyl-glutamine (PAG), (4) short chain fatty acids (SCFAs), and (5) polyphenols [10]. Some of these molecules have been proved to functionally interact with SH, which may explain at least in part, the gender dimorphism in CV risk and CVD outcomes [8].

### 2.1. Bile Acids Modulation and Cholesterol Metabolism

Bile acids (BAs) are considered critical components of the crosstalk between GM and CV health. The GM can directly influence the hepatic regulation of cholesterol metabolism [11] and alter bile acid composition and abundance, which in turn affect systemic cholesterol levels [12]. Originating from cholesterol catabolism, BAs are synthesized in the liver as primary bile acids (PBAs), then conjugated to amino acids taurine or glycine to form bile salts and included in the bile. Next, the bile is secreted in the duodenum to facilitate digestion and absorption of fats, nutrients, and liposoluble vitamins [13]. In the intestine, PBAs are deconjugated by bile salt hydrolase (BSH), an enzyme produced by a broad spectrum of aerobic and anaerobic bacteria (Gram-positive *Bifidobacterium*, *Lactobacillus*, *Clostridium,* and *Enterococcus* and Gram Negative *Bacteroides*). Later, a microbial-mediated dehydroxylation converts PBAs into secondary bile acids (SBAs). This reaction is catalyzed by a limited number of bacteria belonging to different genera (*Bacteroides*, *Clostridium*, *Eubacterium*, *Lactobacillus*, and *Escherichia*) [1]. 

PBAs appear to play a role in preventing the development of atherosclerosis through activation of the farnesoid X receptor (FXR) signaling cascade, which improves lipid profile and regulates gluconeogenesis and intestinal barrier function [14]. In addition, PBAs inhibit the NF-kB-dependent activation of Takeda-G protein 5 receptors (TGR5), resulting in a decreased production of proinflammatory cytokines [10,15]. PBAs can also influence CV function and reduce heart rate by regulating channel conductance and calcium dynamics in sinoatrial and ventricular cardiomyocytes. Finally, PBAs modulate vascular tone through a transcriptional regulation of vasoactive molecules as long-lasting effect [16].

Some studies have demonstrated an association between altered plasma or fecal BAs concentrations and CVD risk [17]. In particular, high concentrations of SBAs have been related with atherosclerosis development and CVD through GM-mediated mechanisms [18]. In fact, BSHs of GM are able to hydrolyze glycine and taurine conjugates to liberate free Bas; the resulting SBAs overproduction promotes enhanced cholesterol levels and increases foam cell formation and atherosclerotic plaque size [15]. Accordingly, a low SBAs excretion represents an independent risk factor for stroke and mortality [17]. Moreover, an alteration of SBAs to PBAs ratio may be implicated in hypercholesterolemia and CAD development. In this regard, Meyerhofer et al. demonstrated that higher plasma levels of SBAs in front of reduced PBAs in heart failure (HF) patients are associated with worse outcomes in univariate analysis [16]. On the other hand, some species of bacteria produce less absorbable SBA molecules that are excreted in the stool. This process stimulates BA neo-synthesis by the liver, leading to a net loss of circulating low-density lipoprotein (LDL) and reduced CV risk [19].

Collectively, the available findings indicate that GM plays a pivotal role in BAs synthesis and in determining the types and amount of PBAs and SBAs, with beneficial or harmful effects on health depending on the composition of the bacteria population.

Another mechanism involving GM in cholesterol metabolism is the conversion of absorbable cholesterol to coprostanol, a reduced non-absorbable sterol [20]. Although further studies are needed to better clarify this pathway, it is conceivable that GM may contribute to lower blood cholesterol and CV risk also by enhancing cholesterol removal through this route. The rate of GM-mediated cholesterol-to-coprostanol conversion in humans is variable: there are high converters showing almost complete cholesterol conversion and low converters with coprostanol representing less than 40% of the fecal neutral sterols content [20]. Strains belonging to the genera of *Eubacterium* and *Bacteroides* have been identified as the prevalent cholesterol-reducing strains [21,22]. Interestingly, coprostanol production is sex-dependent, with young women being better converters compared to young males [23].

### 2.2. Trimethylamine/Trimethylamine N-Oxide (TMAO)

Foods such as meat, eggs, fish, brassica vegetables, peanuts, and soybeans are abundant in choline, betaine, phosphatidylcholine, lecithin, and L-carnitine [24,25,26], which serve as dietary precursors of CM disease-associated compounds. Specifically, these nutrients are converted to trimethylamine (TMA) by particular intestinal bacterial strains endowed with TMA lyases. TMA is subsequently oxidized to trimethylamine N-oxide (TMAO) by the hepatic flavin monooxygenase 3 (FMO3) [27]. TMA producers include: the *Firmicutes Anaerococcus*, *Clostridium*, *Desulfitobacterium*, *Enterococcus*, and *Streptococcus*; the *Proteobacteria Dseulfovibrio*, *Enterobacter*, *Escherichia*, *Klebsiella*, *Proteus*, *Pseudomonas*, the *Actinobacteria Actinobacter*, and *Citrobacter* [28], while *Bacteroidetes* are not TMA producers [29]. Additionally, *Akkermansia*, *Sporobacter*, *Prevotella* [29], and *Ruminococcus Gnavus*, highly represented in GM of patients with atherosclerotic CAD, are great TMAO producers [30].

Noteworthy, the TMAO precursor choline can be also produced directly by GM via phospholipase D (PLD) enzyme, thus confirming the important direct involvement of GM in TMAO production [31].

TMAO represents a risk factor for CAD development as demonstrated by several preclinical and clinical data [26]. In mice, TMAO levels increased upon dietary supplementation of choline and L-carnitine, prompting macrophage foam cell formation and development of atherosclerosis [24,26]. The association of raised TMAO production and adverse CV outcome is also shown in numerous clinical studies [32,33]. In particular, increased TMAO levels have been associated with an elevated risk of fatal and non-fatal myocardial infarction (MI) or stroke [34]. In a large independent clinical cohort (*n* = 4007), patients in the highest quartile of TMAO plasma levels had a 2.5-fold higher risk of a major adverse CV event than patients in the lowest quartile [25]. Moreover, TMAO circulating levels predict 5-year mortality in patients with stable coronary artery disease [33]. 

The mechanisms by which TMAO increases GM-mediated CV risk are numerous. TMAO influences lipid composition [34], alters BAs transport, composition, and pool size, induces C-reactive protein production, fosters endothelial dysfunction, and increases serum levels of the proinflammatory LPS endotoxin [35]. Moreover, TMAO enhances platelet responsiveness to multiple distinct agonists (ADP, thrombin, and collagen) by facilitating Ca^2+^ release from the intracellular stores and inducing a pro-thrombotic effect in vivo [36]. Finally, TMAO promotes platelet aggregation also by activating the toll-like receptor (TLR) pathways [37].

### 2.3. Phenyl-Acetylglutamine (PAG)

Phenyl-acetylglutamine (PAG) is a recently identified, protein-derived compound produced by GM, and is considered a risk factor for atherosclerosis. It is obtained from the conversion of phenylalanine into phenylacetic acid performed by specific strains of GM. Phenylacetic acid is then conjugated with glutamine in the liver, generating phenylaceticglutamine (PAG) [38]. PAG affects platelet responsiveness inducing thrombosis through adrenergic receptor activation [38]. In the Malmo Offspring Study, Ottoson demonstrated that PAG was correlated with GM composition and associated with an increased risk of future CAD independently of other CV risk factors [39].

### 2.4. Short Chain Fatty Acid Production (SCFAs)

Besides transforming the compounds introduced with the diet in pro-atherogenic mediators, GM can also produce metabolites with a protective role.

SCFAs are GM-derived metabolites produced by the fermentation of complex carbohydrates, mostly from fermentable fibers such as pectin, beta-glucans, guar-gum, inulin, and phospho-oligosaccharides, largely contained in fruit, vegetables, and whole grains [40]. The most abundant SCFA is acetate (up to 75% of total SCFAs) followed by propionate and butyrate [41]. Acetate and butyrate are produced by members of the *Bacteroidetes* phylum, whereas butyrate is produced by species of the *Firmicutes* phylum [42]. In the specific case of Firmicutes, most gut bacteria representing this phylum are gram-positive and are able to produce several SCFAs, contributing to the protective CVD phenotype [1].

SCFAs influence numerous processes such as host-microbe signaling, energy utilization, and control of intestinal pH, entailing effects on the GM composition and gut motility [43]. SCFAs contribute to maintaining the intestinal barrier integrity by stimulating the production of mucin and regulating the expression of tight junction proteins [44]. The efficiency of the intestinal mucosa barrier allows a reduction of the leakage of molecules and cells from the gut into the bloodstream and, in turn, participates in reducing the systemic inflammatory condition. Thus, the SCFAs have anti-inflammatory effects [45]. 

SCFAs and, especially butyrate, act on specific plasma membrane receptors to mainly transduce inhibitory effects. For example, the interaction of SCFAs with receptors of immune response cells represses the activation of the nuclear factor NFκB and the activity of histone-deacetylases (HDAC) [46,47]. These two mechanisms are responsible for stopping the proliferation of T lymphocytes, and for inducing apoptosis of activated T lymphocyte when SCFA concentration increases further [48]. The same two mechanisms also blunt the release of TNF-alpha by granulocytes, monocytes, and macrophages after contact with bacterial membrane LPS [49]. In addition, SCFAs activate regulatory T (Treg) cells, through HDAC inhibition, playing a regulatory or suppressive activity on inflammatory signaling [46]. 

Since the SCFA receptors are widespread in many organs and cells such as intestinal epithelium, or adipocytes, the overall action of SCFAs lowers serum lipid levels though several routes: (i) by blocking cholesterol synthesis, (ii) by redirecting lipids towards the liver [50], and (iii) by reducing the triglycerides accumulation in fat cells [51]. Collectively, these SCFA-mediated processes have a protective role against the development of overweight, obesity, and CAD [51]

### 2.5. Polyphenols

Dietary polyphenols are naturally occurring compounds present in food items such as vegetables, fruits, cereals, tea, coffee, dark chocolate, cocoa powder, and wine [52]. The main groups of dietary polyphenols are phenolic acids, flavonoids, tannins, stilbenes, and diferuloylmethanes. The chemical composition of these molecules is extremely variable but presents common domains, i.e., hydroxylated aromatic rings or phenol rings [53]. A large proportion of polyphenols remain unabsorbed along the small intestine and may accumulate in the large intestine (90–95%), where it is extensively metabolized by GM. In turn, the transformed polyphenols have the ability to modify GM growth and composition in a two-way relationship [52]. For example, berberine, a polyphenol with poor oral bioavailability, significantly reduced atherosclerosis in high-fat-diet fed mice by stimulating the growth of *Akkermansia* [54]. Along the same line, the ability of resveratrol to counteract the metabolic syndrome-related alterations, including derangement of glucose and lipid homeostasis, increase of fat mass, rise in blood pressure, low-grade inflammation, and oxidative stress, is mainly due to the regulation of GM composition, BAs biosynthesis, and TMAO production [55]. Furthermore, GM converts polyphenols into secondary metabolites with potential health effects. This is the case of the lignin derivatives, such as enterodiol and enterolactone, that exert protective effects including a 30% reduction of all-cause mortality risk, along with decreased CVD mortality and non-fatal myocardial infarction [56]. 

**Figure 1:**
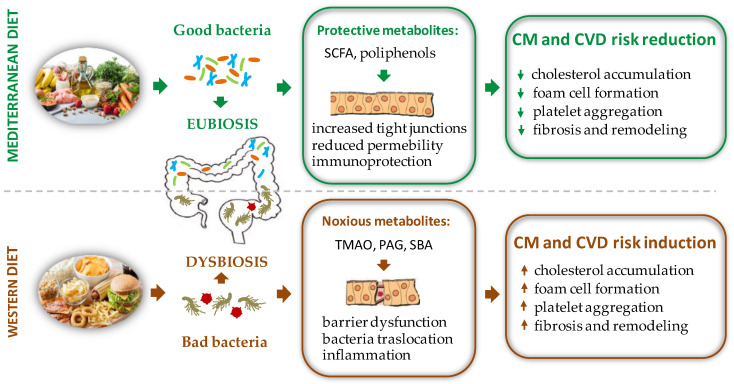
Schematic representation of the effects of different diet regimens on gut microbiota composition with opposite repercussions on CM and CVD risk factors. PAG = phenyl-acetylglutamine, SBA = secondary bile acids, SCFA = short chain fatty acid, TMAO = trimethylamine N-oxide.

Therefore, when compared to a western diet, a polyphenol rich nutritional regimen, such as the Mediterranean one, must be preferred to increase the biodiversity of the GM and ensure advantageous effects for the health of the host (Figure 1).

## 3. Sex Difference and GM Diversity: Bidirectional Cross Talk between Intestinal Microbial Composition and Sex Hormones

In addition to dietary regimen and genetic predisposition, SH, such as progesterone, estradiol, and testosterone, have a significant effect on the regulation of GM abundance and composition. Indeed, males and females have distinct microbial profiles, which highlights the pivotal role of SH levels and types in shaping GM [57,58]. The sex-dependent variability in the GM composition has been assessed both in animal models and in humans [59,60]. In small animals, a substantial gender similarity of GM is observed at a prepubertal age, while sex dimorphism appears at puberty and becomes even more evident in the adult mice. Specifically, females exhibit a relatively higher diversity of GM than males. However, castration restores the analogy between adult male and female GM, thus suggesting that androgens play a key role in inducing such sexual dimorphism [59].

Similarly to rodents, human beings also show gender differences in the GM with men exhibiting a lower microbial diversity than women [60]. Healthy young women show a preponderance of *Firmicutes*, while healthy male subjects show a higher abundance of *Bacteroides Prevotella* than females. Noteworthy GM of post-menopausal women is similar to the male one [61]. This change could contribute to the well-known increase of CV risk in postmenopausal women.

**Figure 2:**
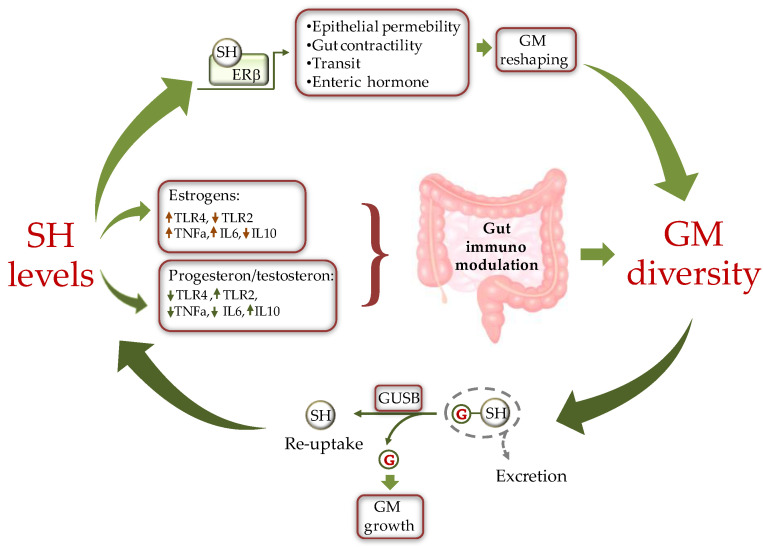
Schematic representation of the cross-talk between sex hormones (SH) and gut microbiota (GM). SH affect GM composition by modulating the intestine milieu via: (i) a direct gene expression regulation in epithelial cells; and (ii) by modulating the gut immune system through differential activation of TLRs. In turn, GM influences SH circulating levels by fine-tuning the balance between hormone excretion and reuptake. ERβ = estrogen receptor beta, GUSB = β-glucuronidases; G = glucuronic moiety, IL = inteleukin, TLR = toll-like receptor.

A bidirectional cross-talk has been proposed to better describe the type of interaction between SH and the GM [61] (Figure 2). According to this paradigm, SH influence intestinal microbial diversity by regulating gene expression, protein production, and other processes of GM. In turn, the microbial population affects SH levels to condition the host physiology and pathophysiology [62]. Such reciprocal GM/SH relationship is involved in the gender-related response to noxious stimuli and could partly explain the gender difference observed in the incidence and progression of certain diseases, including metabolic and CV disease [63]. An intriguing example of how SH and GM interact in the evolution of type-1 diabetes (T1D) derives from investigation on mouse models of non-obese T1D. Exposure of female mice to androgens or the intestinal bacteria of male mice revealed protection against T1D development [64], indicating a critical role of sex-dependent interactions with the GM in the onset of pathological states.

### 3.1. Sex Hormones Regulates GM Composition and Function

Like other members of the steroid hormones superfamily, SH act prevalently through the classical genomic mechanism that involves binding to specific nuclear steroid hormone receptors (SHR) transcription factors, including estrogen receptors (ER), progesterone receptors (PR), and androgen receptors (AR) [65]. Besides the classical mechanism of action, sex steroids can act in the cells through the nonclassical or nongenomic mechanism of action, in most cases mediated by membrane receptors.

Through these pathways, SH may influence GM composition: (1) by a direct action on bacteria, or (2) indirectly, by affecting intestinal PH and motility, and by modifying the function of the intestinal epithelium and local immunological response, thus influencing the environment for bacteria growth.

#### 3.1.1. Direct Effects of Sex Hormones on GM Profile

SH directly affect bacterial growth and induction of virulence factors. In particular, SH, by interacting with ER beta, (ERβ) modulate the bacteria metabolism [66]. For instance, it has long been recognized that the uptakes estradiol and progesterone by the anaerobic bacteria *Prevotella Intermedius*, favors bacterial expansion [67]. In addition, progesterone and estradiol can act as surrogates of vitamin K, an essential growth factor for *Prevotella Intermedius* [62].

Sex steroids could also directly affect the composition of the GM by modifying substrate levels and energy production as the result of changes in bacterial beta glucuronidase (GUSB) activity (Figure 2). Many gut bacteria synthesize GUSB enzymes. These proteins catalyze the release of glucuronic acid from host-derived molecules including SH, previously conjugated in the liver. The released carbon sources are then used by the intestinal bacteria as energy substrate to promote their own growth [68].

#### 3.1.2. Indirect Effects of Sex Hormones on GM Profile

Sex steroids may indirectly modulate the GM growth through activation of specific SHR on the intestinal colonic cells of the host [69]. As a consequence, the intestinal abundance and localization of SHR subtypes is expected to greatly impact GM distribution, composition, and function. 

ERβ is the most abundant ER in colon epithelial cells, followed by ERα. In accordance with its distribution, ERβ is involved in the organization and architectural maintenance of the colon epithelium [70,71]. Indeed, Erβ-knockout mice exhibit a number of pre-pathogenic phenotypes, including disrupted cell-to-cell tight junctions and altered GM compared with wild-type mice, thus indicating a specific physiological role of ERβ in regulating the permeability of colonic epithelia [70,71].

AR is more expressed in human colon rectal mucosa and in colon stromal cells where it contributes to the maintenance of intestinal homeostasis [72]. The modified microenvironment might, in turn, affect the GM composition. 

The classical ligand-dependent regulation of SHR activity by SH in the gastrointestinal cells also influences gut functions such as contractility, transit, or secretion of enteric hormones [73], which in turn induce GM modifications (Figure 2).

Numerous studies have evidenced a close connection between inflammatory state observed in several disease conditions and changes in the composition of GM [74]. SH transcriptional activity influences GM composition by differentially affecting the local intestinal immune environment in a gender-specific way. Estrogens and androgens have opposite effects on the immune response. In particular, estrogens favor a hyperactive immune environment, while androgens (testosterone) have a suppressive effect on T cell proliferation, resulting in an anti-inflammatory condition [75]. On the other hand, progesterone promotes the synthesis of anti-inflammatory cytokines and inhibits the production of their pro-inflammatory counterpart [76].

A major mechanism linking SH, immune response, and GM deals with the integrity of the intestinal epithelial barrier [77]. Decreased cohesion of the gut epithelium is associated with infiltration of gram-negative bacteria into circulation and activation of a peripheral inflammatory response by LPS, which may exasperate existing pathologies such as CM and CV disease [62,64]. The interaction between certain GM strains and estrogens alters the integrity of the intestinal barrier and enhances cell-mediated and humoral immune response, natural killer (NK) cell cytotoxicity, and the production of pro-inflammatory cytokines such as interleukin 1 (IL-1), interleukin 6 (IL-6), and tumor necrosis factor alpha (TNF-α) [77], thus favoring a proinflammatory phenotype. 

As concerns the underlying pathway, in females there is an increased expression of genes involved in the pro-inflammatory TLR pathways. As evidenced by experimental studies, estrogenic treatment in mice increases cell membrane content of TLR4 [78]. Once activated, TLRs induce a pro-inflammatory immune environment that compromises gut permeability, causing translocation of gut commensals into the lamina propria where they can amplify pro-inflammatory responses [62] (Figure 2). In contrast, to estrogens, androgens, such as testosterone, decrease TLR4 and TLR2 expression in macrophage and down-regulate NK cells and TNF-α production while enhancing the production of the anti-inflammatory interleukin 10 (IL-10) [79]. Ultimately, the repression of TLR pathways and antigen presentation by testosterone favors the integrity of the intestinal barrier [79]. Similarly to testosterone, progesterone contrasts the activation of LPS-mediated TLR4 signaling [80] and suppresses NK cell activity, thus fostering the integrity of the intestinal epithelium [62]. Additionally, progesterone decreases gut permeability through upregulating occludin in human intestinal tissue [81].

A recently identified pathway involving SHR, intestinal homeostasis, and GM diversity deals with the activity of the orphan nuclear estrogen-related receptor alpha (ESRRA) on mitochondrial function. This factor is critical to maintain mitochondrial biogenesis and macroautophagy/autophagy function in the gut. It has been demonstrated that ESRRA acts as a key regulator of intestinal homeostasis by ameliorating colonic inflammation through activation of autophagic flux and control of host GM composition. Thus, ESRRA contributes to intestinal homeostasis to protect the host from detrimental inflammation and dysfunctional mitochondria [82].

In conclusion, changes in estrogens, progesterone, or androgens signaling could result in altered intestinal functions, such as contractility and transit, and in a local immune unbalance, which may influence the gender-specific GM composition by differently shaping the gut mucosal environment.

### 3.2. GM Composition Regulates Sex Hormone Levels

Just as GM is influenced by SH, in turn GM may be an important regulator of circulating levels of SH and SH metabolites [63]. In fact, GM is able to produce hormones (e.g., serotonin, dopamine, somatostatine), and to regulate the host’s hormones homeostasis by inhibiting gene transcription (e.g., prolactin) or fostering conversion reactions (e.g., glucocorticoids to androgens) [58]. 

The set of enteric bacterial genes coding for estrogen-metabolizing enzymes, is defined as “estrobolome” [83]. As above anticipated, the bacterial GUSB influences the systemic estrogen metabolite (EM) profiles. Normally, in the phase I metabolic step, estrogens, and their metabolites are conjugated by the liver and excreted in the bile. Of note, glucuronidation of estrogens serves primarily a classical excretory role given that conjugated EM have a low affinity for the ERs and do not promote appreciable transcription. Once secreted within the intestine, EM can be de-conjugated by the GM GUSB, reabsorbed by the gut, and released into the bloodstream for distal action. Thus, depending on GM composition, the estrobolome favors deconjugation and promotes reabsorption of free estrogens into enterohepatic circulation, which in turn contributes to the host’s total estrogen amount [62,64] (Figure 2). When this process is impaired through dysbiosis, the altered deconjugation process results in variation of circulating SH levels. For example, a reduction of bacterial GUSB activity may contribute to the development of pathological conditions such as obesity, metabolic syndrome, cardiovascular disease (CVD), and decline of cognitive function [8,77]. On the other side, the increased abundance of GUSB-producing bacteria can induce hyperestrogenic pathologies. This condition results in increased free estrogens circulating levels, entailing diseases such as endometriosis and cancer [84,85].

Increasing findings also indicate that male androgen levels are subjected to GM-dependent modulation. In studies on obese male mice, the treatment with lactic acid bacteria isolated from human milk induces growth of testes and increased serum testosterone levels [86]. In addition, feeding the old male mice with purified microbes such as *Lactobacillus Reuteri* restores the testosterone to the youthful level [87]. Collectively, these results provide evidence that GM is able to affect testosterone production and testicular aging. 

Although human androgen biosynthesis and metabolism have been extensively studied in the past, the exact underlying mechanisms still remain elusive. Traditionally, most androgen has been thought to be metabolized by the liver and subsequently excreted by the kidney [88]. However, a recent study by Collden et al. pointed at the GM as a main regulator of androgen metabolism [89]. In effect, similarly to estrogens, conjugated androgens are hydrolyzed in the intestinal tract via bacterial GUSB into free androgens for reabsorption. A reduced concentration of circulating androgen may induce androgen-related disease. As reported by Harada et al., the GM was largely involved in the CV risks associated with male hypogonadism, which could be prevented by antibiotic therapeutic strategies [90,91]. In line with this notion, in a recent post hoc analysis, men with metabolic syndrome and low levels of high-density lipoprotein (HDL)-cholesterol evidenced an association between reduced testosterone concentrations and increased risk of subsequent CV events and death [92]. This finding is confirmed by other epidemiological studies showing that low testosterone levels in men are negatively associated with the degree of carotid atherosclerosis [60]. Finally, it is well-established that male hypogonadism in aging men is closely associated with an increased risk of metabolic syndrome and T2D [59]. 

Besides the androgen deglucuronidation activity of GM, some specific microbial taxa, including *Butyricicoccus Desmolans*, *Clostridium Cadaveris*, *Propionimicrobium Lymphophilum*, *Clostridium Scindens*, and *Clostridium Innocuum*, express classical steroid processing enzymes such as steroid-17, 20-desmolase, 20β-, 20α-, and 3α-hydroxysteroid dehydrogenase, or 5β-reductase, and possess the potential capability to directly metabolize steroid hormones [93,94]. In addition, glucocorticoids can be converted into androgens via side-chain cleaving capacity of bacteria [95,96]. Finally, to make the scenario of interactions between GM and SH even more complex, a recent work has shown the ability of some bacterial strains to retroconvert estrogens into androgens [97].

Overall, the present evidence underlines a critical role of GM in estrogens and androgen metabolism, which deserves to be explored in more detailed studies.

## 4. Role of the GM/Sex Hormone Axis in Metabolic and Cardiovascular Disease Risk Factors

CVD is responsible for a large incidence of death cases in both men and women worldwide [98]. Even though age-adjusted CVD mortality rates are higher in men compared to premenopausal women, the midlife period, coincident with the menopause transition, leads to a significant increase of CHD risk factors also in women [99].

Many studies have demonstrated the association between specific GM signature and several CVD manifestations, highlighting the potential roles of bacteria in the pathogenesis of coronary heart disease, and CM disorders [100,101,102]. Unfortunately, most studies linking GM dysbiosis to CVD risk factors and focused on gender difference were performed in preclinical mouse models rather than in humans. For example, in experimental studies, ovariectomy and castration allowed to evaluate the hormonal impact on GM and susceptibility to disease [103]. In hypertensive animals, dysbiosis and decreased *Bacteroidetes* to *Firmicutes* ratio was observed [104]. In diet-induced obese mice, *Akkermansia Muciniphila* abundance was strongly correlated with lipid metabolism and inflammation markers in adipose tissue [105]. In the clinical settings, the correlations between SH and GM composition and their role in disease development and progression have been described in separate studies. Few papers, focused primarily on irritable bowel syndrome and autoimmune diseases, have performed differential, gender-specific analyses to explore how the reciprocal interaction between GM diversity and SH affects the onset and development of these pathologies [106,107]. There is an urgent need of new studies investigating the development of CDV and MS and considering the GM/SH cross talk as a critical pathological trigger. The knowledge of these connections could be fundamental to increase our understanding of disease onset and progression and identify new targets for therapeutic purposes.

### 4.1. Metabolic Syndrome and Diabetes

Although the field is still young, sex differences in the GM composition are regarded as key determinants of gender predisposition to metabolic syndrome and diabetes mellitus (DM). Indeed, dyslipidemia, dysglycemia, obesity, and DM may all induce GM alterations and, vice-versa, GM may affect the onset and evolution of metabolic syndrome [108]. For example, in an animal model of high fat diet (HFD)-induced metabolic disorders, body weight gain and insulin resistance were higher in male than in female mice [109]. Antibiotic-pretreatment alleviated diet-induced insulin resistance in male mice while increasing fasting blood glucose in females. The different metabolic responses to HFD were paralleled by a remarkable dimorphism in GM composition with a higher abundance of the genera *Parabacteroides*, *Lactobacillus*, *Bacteroides*, and *Bifidobacterium* observed in females than in males. Importantly, HFD remodeled GM by decreasing the abundance of the protective SCFA-producing bacteria such as *Roseburia* and *Lachnospiraceae* group. Additionally, a gender bias in GM composition following antibiotic pretreatment in HFD mice was observed, thus indicating a sex-dependent sensitivity to antibiotics [109].

On the other hand, androgen deficiency in males can promote metabolic disorders in the presence of an HFD. Androgen deprivation via castration altered fecal microbiota and exacerbated risk factors for CVD, including obesity, fasting glucose, and hepatic triglyceride accumulation [91]. GM depletion through antibiotics treatment alleviated the metabolic alterations induced by hypogonadism [90].

**Figure 3:**
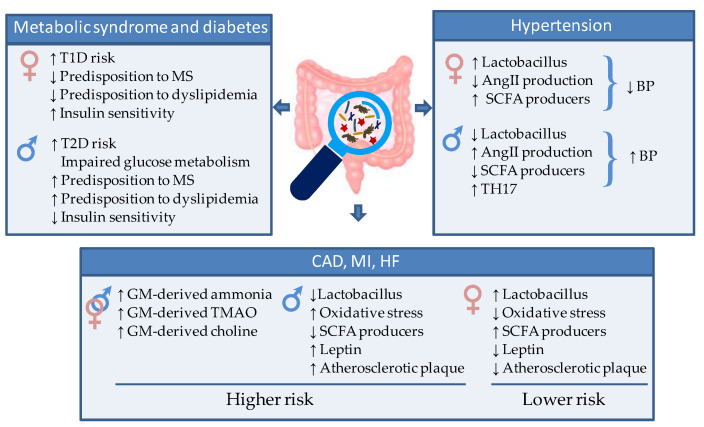
Schematic representation of the gut microbiota-mediated sex differences in CM and CV risk and disease. AngII = Angiotensin 2. SCFA = short chain fatty acid, TH17 = T helper 17 cells, TMAO = Trimethylamine/trimethylamine N-oxide, T1D = type 1 diabetes, T2D= type 2 diabetes.

Generally, males are more susceptible than females to impaired glucose metabolism and type 2 diabetes (T2D) [110]. In this context, a recent experimental study revealed that the sex-dependent difference in glucose metabolism, commonly observed in both humans and rodent animals, depends on a different shaping of GM. Mechanistically, this work demonstrated a key role of androgens in deteriorating glucose homeostasis by modulating the GM composition and the circulating levels of glutamine and glutamine/glutamate ratio, thereby contributing to the difference in glucose metabolism between the two sexes [111] (Figure 3).

While the obese T2D is more commonly diagnosed in males, the non-obese type I diabetes mellitus (T1D) is observed predominantly in females [60]. It has been shown that fecal microbiota transfer from young male mice to females significantly improved the serum testosterone in the recipients and conferred them an acquired resistance to T1D [75]. This interesting finding suggested that male GM is able to modulate androgen in females and protect them from T1D. 

In line with experimental models, in human beings, several studies have indicated differences in the GM as potential determinants of gender predisposition to metabolic syndrome, insulin resistance, and diabetes [112] (Figure 3). For instance, T2D is associated with a reduction of the protective butyrate-producing species, and an increase in *Bacteroides–Prevotella* species [113,114,115], while transfer of intestinal microbiota from lean donors increases insulin sensitivity in individuals with metabolic syndrome [116]. A recent clinical trial (CARDIOPREV NCT00924937) provides evidence of a different GM composition in patients with metabolic syndrome according to gender and a different shaping of GM after 3-year consumption of a Mediterranean or a low-fat diet (LFD). Women evidenced higher levels of *Collinsella*, *Alistipes*, *Anaerotruncus*, and *Phascolarctobacterium* genera, whereas the abundance of *Faecalibacterium* and *Prevotella* genera was higher in men. Moreover, elevated levels of *Desulfovibrio*, *Roseburia,* and *Holdemania* were observed in men than in women after the consumption of the LF diet [112].

Insulin resistance represents an underlying mechanism of the dysmetabolic syndrome frequently associated with GM dysbiosis (Figure 3). In combination with the activity of SH, an altered intestinal bacteria composition could exacerbate insulin resistance via LPS-mediated modulation of the TLR signaling. For example, alteration of TLR2 pathways and activation of TLR4 by estrogens potentiated the serum LPS-induced inflammatory signals in macrophages leading to an impairment of insulin signaling in the muscle, liver, and adipose tissue [117]. The importance of metabolic endotoxemia in the onset of insulin resistance has been demonstrated in TLR knockout animal models. Mice lacking TLR4 were protected against HFD-induced insulin resistance [118]. This mechanism has been confirmed also in TLR2 knockout mice in association with higher proportions of *Bacteroidetes* and *Firmicutes* coupled with a lower proportion of *Proteobacteria* phyla [119]. On the contrary, progesterone, thanks to its anti-inflammatory action, appears to exert a protective effect against the LPS-dependent impairment of insulin signaling [62].

Another route through which the GM influences risk factors in relation to CM disorders is by regulating the homeostasis of the hormones of the gastro-intestinal system. Human studies have demonstrated that GM re-shaping with fermentable fibers increases SCFAs-producers and exerts beneficial effects by modifying the production and plasma levels of enteroendocrine hormones involved in appetite sensation and glucose response (i.e., glucagon-like peptide 1, peptide YY, and Ghrelin) [120,121]. Along the same line, it has recently been demonstrated that probiotics affecting GM composition improve fasting glycaemia, hyperinsulinaemia, insulin resistance index (HOMA-IR), and glycated hemoglobin via gut peptides secretion [122,123]. 

In addition to influencing the onset and progression of the disease, the GM can affect the response to pharmacological treatments in a gender-specific way. For example, pioglitazone, a peroxisome proliferator-activated receptor gamma (PPAR-γ) agonist used as hypoglycemic drug in the management of T2D, shows a stronger efficacy in female with respect to male mice [124,125]. One of the supposed mechanisms of this gender difference is related to an upregulation of the PPAR-γ receptor mediated by the estrogen 17β-estradiol. This finding is in accordance with the increased susceptibility to dyslipidemia observed in men compared to women [109].

### 4.2. Hypertension

Hypertension is a major modifiable risk factor for CVD. GM dysbiosis has been demonstrated both in animal models and in hypertensive populations [126,127]. Compared to individuals with normal blood pressure (BP), essential hypertensive patients show intestinal epithelial barrier dysfunction and altered GM composition [128], which can be prevented with probiotic *Lactobacillus* or antibiotic treatment [129]. Additionally, the fecal microbiota transplantation (FMT) from hypertensive patients to germ-free mice induced BP elevation, thus demonstrating a direct connection between GM alteration and hypertension [130]. Based on this evidence, an ongoing clinical trial is dedicated to establish the safety and efficacy of bacteriotherapy through FMT from healthy donors to hypertensive patients [131].

GM-host interaction affects BP through multiple routes. One main mechanism involves the crosstalk with the nervous system. For example, enhanced sympathetic drive has been reported to contribute to a shift in gut microbial genera, resulting in increased permeability of gut epithelial barrier, local gut inflammation, and BP elevation [126]. In addition, GM products are implicated in sympathetic activation. In particular, some bacteria of *Streptococcus*, *Escherichia*, *Lactobacillus*, and *Bifidobacterium* genera can produce neuroactive compounds that affect the vascular tone via modulation of the autonomic nervous system [132]. Alteration in the prevalence of these bacteria contributes to the development of hypertension. [133]. For example, in a rat model, the hypertensive state was paralleled by a reduction in GM *Latobacilli* and could be ameliorated by exogeneous probiotic administration [134]. The pressure-lowering activity of *Lattobacilli* is mainly mediated by the secretion of peptides that inhibit angiotensin-converting enzyme, leading to decreased production of angiotensin II, a strong vasoconstrictor [135,136]. It has been demonstrated that women have higher levels of *Lactobacilli* in the gut [137,138], which may partly explain the lower pressure levels in fertile women compared to age-matched men. Consistently, men show greater blood pressure rises than women in response to angiotensin II [139] (Figure 3). 

Bacteria of the *Lactobacillus* and *Bifidobacter* genera play a protective role also via the pressure-lowering effects of SCFA metabolites, whose production is inversely associated with hypertensive conditions [140] (Figure 3). A low abundance of the high SCFA-producer *Bifidobacteria* has been demonstrated in GM of hypertensive rats [104]. On the other hand, administration of antibiotics, high-fiber (prebiotic) diet, or probiotics to increase SCFA-producing bacteria was effective in reducing both systolic and diastolic BP [135,141]. The anti-hypertensive effects of GM-derived SCFA are due in part to their influence on vascular tone and renal sensory nerves [140], and in part to the above-described anti-inflammatory action. Indeed, chronic low-grade inflammation, favored by GM dysbiosis, contributes to a raise in BP [142]. GM-driven proinflammatory state, aggravated by impairment of the renine/angiotensine system and unbalanced salt regulation, can induce endothelial dysfunction ultimately contributing to hypertension. Human studies have demonstrated that the abundance of *Faecalibacterium*, *Bifidobacterium*, *Ruminococcus,* and *Prevotella* is inversely correlated to different low-grade inflammation markers that have impact on host blood pressure such as high sensitivity C-reactive protein and IL6 [143]. The antihypertensive responses induced by gut-derived SCFA-delivers are partly explained by the anti-inflammatory effects of propionate [144] and may reveal GM contributions to sex differences in hypertension. T helper (TH) 17 cells (TH17), activated by action of certain microbiota strains, are key pro-inflammatory protagonists correlated to hypertensive state [145,146] (Figure 3). Hypertensive male rats present more TH17 cells compared to female rats in association with reduced SCFA-producer *Lactobacilli* [147,148], thus supporting the relevance of GM/SH axis in setting of hypertension. In the human beings, the reduced GM diversity observed in men can lead to low-grade inflammation contributing to hypertension development. Conversely, estrogens can reduce inflammation concurring to the maintenance of normal BP values during the fertile age.

Finally, arterial stiffness is an independent factor of CV risk particularly relevant to women and is closely related to hypertension. In the post-menopause period, the vascular stiffness increases in parallel with BP. Menni et al. studied the role of GM in arterial stiffness in women, evidencing, for the first time, that GM composition is strongly correlated with levels of arterial stiffness independently of visceral fat and other obesity-related traits, thus suggesting that targeting the microbiome may be a way to treat arterial ageing [149].

Collectively, these data suggest a strong association between GM dysbiosis, hypertension, and gender. However, further clinical studies investigating the impact of the gut microbiome on sex differences in BP and hypertension are necessary. 

### 4.3. Atherosclerosis, Myocardial Infarction and Heart Failure

Clear sex differences have been documented in the onset and evolution of atherosclerosis and CVD [150] (Figure 3). On average, women show reduced atherosclerotic plaque than men at any age and experience ischemic events, such as myocardial infarction (MI), at a later age. A main mechanism for this sex bias is oxidative stress (OS), which is considered a key trigger of coronary artery disease. Pre-menopause women show lower levels of OS than men, due to the antioxidant properties of estrogens, which up-regulate the expression and protein content of anti-oxidant enzymes, including nicotinamide adenine dinucleotide phosphate (NADPH)-oxidase and angiotensin II [151] (Figure 3). In agreement, a recent proteomic profiling revealed sexual dimorphism of the protein content in human atherosclerotic tissue. The main differences concerned response to oxygen species and inflammatory signaling, along with complement activation, and blood coagulation [152]. Sex-related differences have also been reported in the association between non-calcified or mixed coronary atherosclerotic plaques and plasma fibrinogen, a well-known risk factor for CVD [153].

Several studies confirm the relationship between GM dysbiosis and the pathogenesis of atherosclerosis, MI, coronary artery disease (CAD), and HF in humans. 

A major mechanism through which GM dysbiosis favors CVD evolution in patients, is through the production of irritating or noxious molecules. For example, excessive GM-derived ammonia and ammonium hydroxide results in a local pro-inflammatory state that disrupts the intestinal epithelial tight junctions and may propagate at a systemic level (Figure 3). Of note, the identification of bacterial DNA, mostly *Proteobacteria*, both in the atherosclerotic lesions and in the gut of the same individuals [100] points at a key role of a leaky gut epithelial barrier in the translocation of gut bacteria to the site of plaque formation. Further, high levels of circulating TMAO produced by GM have been found in association with vulnerable coronary plaque and plaque rupture and represent a relevant long-term risk of incident CV events in patients with acute coronary syndrome [154,155]. In a study of more than 1800 stable cardiac patients undergoing elective coronary angiography, all TMAO-associated metabolites were positively correlated with prevalent CVD and incident CV events. Additionally, a follow-up study showed that TMAO is a predictive biomarker of all-cause mortality or reinfarction at 2 years after MI [138]. Therefore, TMAO can be considered a critical participant in enhanced risk for atherosclerosis and MI (Figure 3). 

Another mechanistic link between altered GM diversity and the severity of MI has been reported in rats [156]. High systemic leptin levels represent a well-known risk factor of MI and CVD (Figure 3). Administration of *Lactobacillus Plantarum* suppresses circulating leptin, and improves left ventricular function, ultimately leading to decreased myocardial infarct size and post ischemic adverse chamber remodeling [150,157]. Interestingly, experimental studies have indicated a greater abundance of *Lactobacillus Plantarum* in females than in males [158], which can contribute to the gender difference observed in MI and related CVD. Finally, GM has been involved in increased availability of different metabolites of the aromatic amino-acids that are associated with augmented severity of induced MI [159].

HF is a devastating disease with high morbidity and mortality [160]. GM and their dietary-derived metabolites have been implicated in the so-called “gut hypothesis of HF”. In effect, gut dysbiosis, with decreased microbial richness, enhanced inflammation and increased gut permeability, has been observed in conjunction with decreased cardiac output and elevated systemic congestion either in mouse models of pressure overload-induced HF and in HF patients [161,162,163].

Available experimental findings have evidenced a causal role of TMAO in systolic dysfunction and adverse remodeling of HF, including myocardial hypertrophy, wall thinning, ventricular dilation, and increased fibrosis [164,165]. Consistently, clinical studies have confirmed that the GM-derived metabolites TMAO and choline are increased in HF and are associated with poor prognosis even after adjusting for traditional risk factors [11,166,167,168,169]. Collectively, these data suggest a key role of GM and their metabolites in the pathogenesis and progression of HF. In turn, reduced intestinal blood flow, due to heart functional impairment, leads to GM re-shaping with increased bacterial mass and reduced diversity in colonic mucosa, and decreased microbial charge in the stool [11,163]. 

At present there are no data reporting the effect of GM dysbiosis on the development of HF in relation to gender dimorphism.

## 5. Conclusions and Future Perspectives

Gut microbiome-host interaction is of paramount relevance in health and disease susceptibility. Via its multiple roles in metabolizing dietary components, influencing circulating SH levels and inflammation state, GM plays a key role in CM and CVD and represents a driving force of the observed sexual dimorphism in CVD onset and progression. Endogenous SH and GM influence each other; the hormonal milieu modulates the composition and diversity of GM which, in turn, influences the metabolism of sex hormones with repercussions on CM and CVD risk factors. Despite its relatively early introduction in 2013, the concept of microgenderome, intended as the interaction between GM, SH, and immunity [170], has not been exhaustively framed in the context of CM and CV disease, especially in the clinical arena. Though preclinical studies have clearly demonstrated the involvement of microbiome-dependent bile acid metabolism, toll-like receptor signaling cascades, steroid hormone modulation, and immune response as important drivers in sex differences in CVD risk, human studies are still lacking. The literature reviewed herein, while unveiling novel molecular pathways mainly emerged from animal models, highlights the critical importance of innovative human studies specifically designed to assess the role of GM, SH, and inflammation as cross-talking players in the onset and progression of CM and CV disease. In particular, a better understanding of the intestinal barrier function in relation to sex, age, and GM diversity may unveil the role of GM in triggering key sex-specific biological determinants of CVD. 

Further human mechanistic evidence should pave the way for potential low risk, personalized interventions aimed at reducing CM and CVD risk by targeting GM.

## Data Availability

Not applicable.
